# Metabolome analysis-based design and engineering of a metabolic pathway in *Corynebacterium glutamicum* to match rates of simultaneous utilization of d-glucose and l-arabinose

**DOI:** 10.1186/s12934-018-0927-6

**Published:** 2018-05-17

**Authors:** Hideo Kawaguchi, Kumiko Yoshihara, Kiyotaka Y. Hara, Tomohisa Hasunuma, Chiaki Ogino, Akihiko Kondo

**Affiliations:** 1 Graduate School of Science, Technology and Innovation, Kobe University, 1-1 Rokkodai, Nada, Kobe, 657-8501 Japan; 2 Department of Environmental and Life Sciences, School of Food and Nutritional Sciences, University of Shizuoka, 52-1 Yada, Suruga, Shizuoka, 422-8526 Japan; 3 Department of Chemical Science and Engineering, Graduate School of Engineering, Kobe University, 1-1 Rokkodai, Nada, Kobe, 657-8501 Japan; 4 Biomass Engineering Research Division, RIKEN, 1-7-22 Suehiro, Turumi, Yokohama, Kanagawa 230-0045 Japan

**Keywords:** *Corynebacterium glutamicum*, Simultaneous utilization, l-Arabinose, Metabolic engineering, Metabolome analysis

## Abstract

**Background:**

l-Arabinose is the second most abundant component of hemicellulose in lignocellulosic biomass, next to d-xylose. However, few microorganisms are capable of utilizing pentoses, and catabolic genes and operons enabling bacterial utilization of pentoses are typically subject to carbon catabolite repression by more-preferred carbon sources, such as d-glucose, leading to a preferential utilization of d-glucose over pentoses. In order to simultaneously utilize both d-glucose and l-arabinose at the same rate, a modified metabolic pathway was rationally designed based on metabolome analysis.

**Results:**

*Corynebacterium glutamicum* ATCC 31831 utilized d-glucose and l-arabinose simultaneously at a low concentration (3.6 g/L each) but preferentially utilized d-glucose over l-arabinose at a high concentration (15 g/L each), although l-arabinose and d-glucose were consumed at comparable rates in the absence of the second carbon source. Metabolome analysis revealed that phosphofructokinase and pyruvate kinase were major bottlenecks for d-glucose and l-arabinose metabolism, respectively. Based on the results of metabolome analysis, a metabolic pathway was engineered by overexpressing pyruvate kinase in combination with deletion of *araR*, which encodes a repressor of l-arabinose uptake and catabolism. The recombinant strain utilized high concentrations of d-glucose and l-arabinose (15 g/L each) at the same consumption rate. During simultaneous utilization of both carbon sources at high concentrations, intracellular levels of phosphoenolpyruvate declined and acetyl-CoA levels increased significantly as compared with the wild-type strain that preferentially utilized d-glucose. These results suggest that overexpression of pyruvate kinase in the *araR* deletion strain increased the specific consumption rate of l-arabinose and that citrate synthase activity becomes a new bottleneck in the engineered pathway during the simultaneous utilization of d-glucose and l-arabinose.

**Conclusions:**

Metabolome analysis identified potential bottlenecks in d-glucose and l-arabinose metabolism and was then applied to the following rational metabolic engineering. Manipulation of only two genes enabled simultaneous utilization of d-glucose and l-arabinose at the same rate in metabolically engineered *C. glutamicum*. This is the first report of rational metabolic design and engineering for simultaneous hexose and pentose utilization without inactivating the phosphotransferase system.

**Electronic supplementary material:**

The online version of this article (10.1186/s12934-018-0927-6) contains supplementary material, which is available to authorized users.

## Background

l-Arabinose is the second most abundant component of hemicellulose in lignocellulosic biomass, next to d-xylose. Lignocellulosic feedstocks contain cellulose and hemicellulose (14.3–49.9 and 8.8–22.4%, respectively) [1]. Hydrolysis of the lignocellulosic feedstocks yields d-glucose, d-xylose, l-arabinose, and other minor sugar. In the hydrolysates, d-xylose (12–23%) and d-glucose (2–24%) are typically abundant components, followed by l-arabinose (2–6%), although the composition of these monosaccharides depends on feedstocks and hydrolysis conditions [2–4]. In some cases, l-arabinose-rich hydrolysate was also reported. For instance, corn fiber hydrolysate consists of 22% l-arabinose 38% d-glucose, and 30% xylose [5], and sugar beet pulp hydrolysate consists of 78% l-arabinose, 3.9% d-glucose, and 1.4% d-xylose [6]. Thus, the capability of metabolizing these pentoses and d-glucose simultaneously is important for optimal microbial fermentation to more efficiently utilize carbohydrates contained in lignocellulosic feedstocks, as the hydrolysates are predominantly composed of hexose(s) and pentose(s) [7]. However, few microorganisms are capable of utilizing pentoses for bio-based production.

**Fig. 1:**
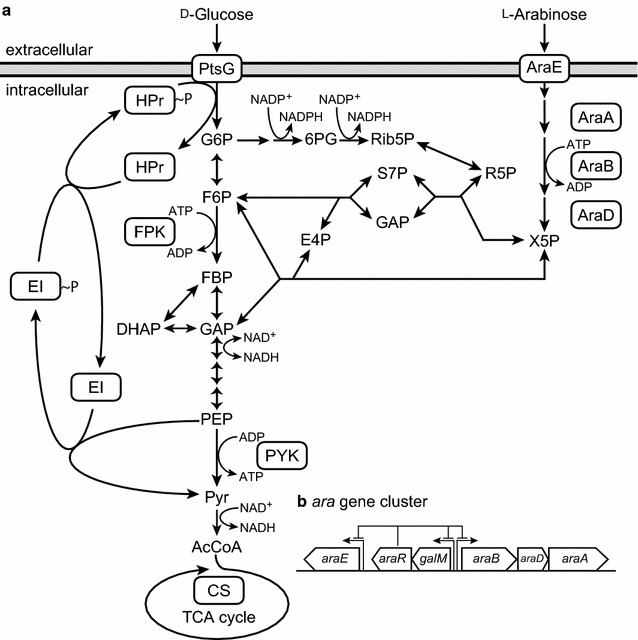
Metabolic pathway of d-glucose and l-arabinose (**a**) and the chromosomal gene cluster regulating l-arabinose utilization (**b**) in *Corynebacterium glutamicum* ATCC 31831

*Escherichia coli* expresses specific transporters and metabolic enzymes that enable the bacteria to utilize both d-xylose and l-arabinose [8, 9]. To take up l-arabinose, *E. coli* expresses two transporters, a low-affinity H^+^ symporter and a high-affinity adenosine 5′-triphosphate (ATP)-dependent transporter encoded by *araE* and *araFGH*, respectively [10, 11]. Once taken up by cells, l-arabinose is converted to l-ribulose, l-ribulose 5-phosphate, and d-xylulose 5-phosphate (X5P) in reactions catalyzed by l-arabinose isomerase (encoded by *araA*), l-ribulokinase (*araB*), and l-ribulose-5-phosphate 4-epimerase (*araD*), respectively [9]. X5P then enters the non-oxidative pentose phosphate pathway (PPP) (Fig. 1).

Catabolic genes and operons enabling bacterial utilization of pentoses are typically subject to carbon catabolite repression (CCR) by more-preferred carbon sources, such as d-glucose [12]. This distinct regulatory system hinders the simultaneous utilization of d-glucose and pentoses such as d-xylose and l-arabinose. Considerable research has focused on inactivation of the phosphotransferase system (PTS) that regulates glucose uptake and metabolism [13] to avoid CCR of pentose metabolism, particularly in *E. coli* [14]. However, inactivation of the PTS markedly reduces d-glucose utilization [15]. After PTS inactivation, adaptive evolution selection of PTS^−^ Glc^+^ mutants has been reported to restore d-glucose metabolism [16], however this can be negatively impacted by the accumulation of undesirable spontaneously arising mutations in the host genome. Thus, an alternative and more rational strategy is needed to develop a microbial platform for the simultaneous utilization of d-glucose and pentose(s).

*Corynebacterium glutamicum* is a non-pathogenic Gram-positive bacterium widely studied for the production of amino acids [17] and a variety of commodity chemicals [18, 19]. However, unlike *E. coli*, *C. glutamicum* lacks pentose assimilation pathways [20], with the exception of strain ATCC 31831, which has a gene cluster comprised of *araBDA*, *araE*, and the transcriptional regulator encoded by *araR* (Fig. 1) and grows on l-arabinose as the sole carbon source [21]. Metabolic engineering of *C. glutamicum* to express *araBAD* from *E. coli* or *xylAB* enables the organism to catabolize the pentoses l-arabinose and d-xylose, respectively [22–29]. Moreover, integration of the Weimberg pathway from *Caulobacter crescentus* enables the efficient utilization of d-xylose [30–32]. However, simultaneous utilization of d-glucose and pentose(s) is possible only in culture at a relatively low concentration of d-glucose [33] or in a high-cell-density reaction [27, 34], or following metabolic engineering to produce a PTS^−^ mutant, resulting in reduced d-glucose utilization [15, 35, 36]. These limitations could be overcome by the rational design of a metabolic pathway enabling the simultaneous utilization of multiple sugars at varying proportions.

In the present study, metabolome analysis was employed to identify the bottlenecks limiting utilization of either d-glucose or l-arabinose by *C. glutamicum* ATCC 31831. Based on this analysis, a pathway for simultaneous utilization of d-glucose and l-arabinose was rationally designed and engineered. Elimination of the major bottleneck limiting l-arabinose metabolism enabled the simultaneous and complete utilization of d-glucose and l-arabinose at the same rate by the metabolically engineered strain, with minimal decline in d-glucose consumption. The metabolome analysis also revealed contrasting metabolic profiles of preferential and simultaneous utilization of d-glucose and l-arabinose. This is the first report of the application of metabolome analysis in the design and evaluation of an engineered pathway for simultaneous hexose and pentose utilization, resulting in bypass of CCR without inactivating the PTS that markedly reduces d-glucose utilization.

## Methods

### Bacterial strains, media, cultivation conditions, and plasmids

Bacterial strains and plasmids used in this study are listed in Table 1. For genetic manipulations, *E. coli* strains were grown at 37 °C in Luria–Bertani medium [37]. For pre-cultivation of *C. glutamicum*, nutrient-rich A medium [38] supplemented with d-glucose (40 g/L) was used, unless indicated otherwise. For cultivation of *C. glutamicum*, mineral salt BT medium [38] supplemented with d-glucose and l-arabinose (15 g/L each) was used, unless indicated otherwise. Broth cultures of *C. glutamicum* were incubated at 30 °C in 500-mL baffled flasks with constant agitation (180 rpm). Where appropriate, media were supplemented with kanamycin (50 µg/mL) for both *E. coli* and *C. glutamicum*.

**Table 1:** Strains and plasmids used in this study

Name	Relevant characteristics	Reference or source
Strain		
*Escherichia coli* JM109	*recA1 endA1 gyrA96 thi hsdR17*(r_K_^−^ m_K_^+^) *e*14^−^ (*mcrA*) *supE44 relA1* Δ(*lac*-*proAB*)/F’ [*traD36 proAB*^+^ *lacI*^q^ *lacZ*ΔM15]	TakaraBio
*Corynebacterium glutamicum* ATCC13032	Type-strain of *C. glutamicum*	ATCC
*C. glutamicum* ATCC31831	Wild type	ATCC
Δ*araR*	Markerless *araR*-deletion mutant of strain ATCC31831	This study
31831/pCH	*C. glutamicum* ATCC31831 bearing pCH	This study
31831/pCH*pyk*	*C. glutamicum* ATCC31831 bearing pCH*pyk*	This study
Δ*araR*/pCH	Δ*araR* bearing pCH	This study
Δ*araR*/pCH*pyk*	Δ*araR* bearing pCH*pyk*	This study
Plasmid		
pCH	Km^r^; *E. coli*–*Corynebacterium* sp. shuttle vector	[40]
pCH*gltA*	Km^r^; pCH with PCR fragment containing *C. glutamicum* ATCC31831 *gltA* gene (NCgl0795) encoding citrate synthase	
pCH*pfk*	Km^r^; pCH with PCR fragment containing *C. glutamicum* ATCC31831 *pfk* gene (NCgl1202) encoding phosphofructokinase	
pCH*pyk*	Km^r^; pCH with PCR fragment containing *C. glutamicum* ATCC31831 *pyk* gene (NCgl2008) encoding pyruvate kinase	This study
pK19mobsacB	Kan^r^, mobilizable *E. coli* vector for the construction of insertion and deletion mutants of *C. glutamicum* (*oriV*, *sacB*, *lacZ*α)	ATCC
pK19mobsac-Δ*araR*	Kan^r^, pK19mobsacB with the deletion construct for *araR* of *C. glutamicum* ATCC31831	This study

### DNA manipulation

All restriction endonucleases were purchased from New England Biolabs (Ipswich, MA). PrimeSTAR Max DNA Polymerase (TakaraBio, Shiga, Japan) was used in PCR to amplify DNA fragments, according to the manufacturer’s instructions. PCR fragments were purified using a QIAquick PCR Purification kit (Qiagen, Hilden, Germany). Electroporation was used to transform *C. glutamicum*, as previously described [39], whereas *E. coli* was transformed using the CaCl_2_ procedure [37]. Plasmid DNA was isolated from *E. coli* as previously described [22].

### Construction of recombinant *C. glutamicum* strains

The *E. coli*–*C. glutamicum* shuttle vector pCH [40] was used to overexpress *gltA* (NCgl0795), *pfk* (NCgl1202), and *pyk* (NCgl2008) genes encoding *C. glutamicum* ATCC 13032 citrate synthase, 6-phosphofructokinase, and pyruvate kinase, respectively. DNA fragments encoding the *gltA* and *pyk* genes were amplified by PCR using the *C. glutamicum* ATCC 13032 genome as the template and oligonucleotide primer pairs (primers 1 and 2 and primers 3 and 4, respectively; Table 2). The resulting two PCR amplicons were digested with *Bam*HI and *Pst*I and subsequently ligated into *Bam*HI and *Pst*I-digested pCH DNA, generating the constructs pCH*gltA* and pCH*pyk*, respectively (Table 1). Likewise, a DNA fragment encoding the *pfk* gene was amplified by PCR using the *C. glutamicum* ATCC 13032 genome as the template and oligonucleotide primers 5 and 6. The resulting PCR amplicons were digested with *Bam*HI and *Sac*I and subsequently ligated into *Bam*HI and *Sac*I-digested pCH DNA, generating the construct pCH*pfk* (Table 1).

Strains of *C. glutamicum* with a deleted *araR* repressor gene were constructed as previously described [41]. Regions upstream and downstream of *araR* were amplified by PCR using the *C. glutamicum* ATCC 31831 genome as the template and oligonucleotide primer pairs 7 and 8 and 9 and 10, respectively (Table 2). The PCR-amplified upstream and downstream fragments were digested with the restriction enzyme pairs *Pst*I and *Hin*dIII and *Pst*I and *Eco*RI, respectively. The *Pst*I and *Eco*RI-digested downstream fragment was ligated into *Pst*I and *Eco*RI-digested pK19mobsacB (American Type Culture Collection, Manassas, VA). The resulting plasmid was digested with *Pst*I and *Hin*dIII and then ligated with the *Pst*I and *Eco*RI-digested upstream fragment, generating the construct pK19mobsac-Δ*araR* (Table 1). Gene deletion was confirmed by PCR using oligonucleotide primers 7 and 10.

**Table 2:** Oligonucleotides used in this study

Name	Target gene	Sequence (5′-3′)	Cohesive ends^a^
Primer 1	*gltA*	CTCTGGATCCGACTACTTCCGTAATCCGGA	*Bam*HI
Primer 2	*gltA*	CTCTCTGCAGCCGGTAGCTCAATCTGTGGC	*Pst*I
Primer 3	*pyk*	CTCTGGATCCATGGTAGTACCTGTGGCTTG	*Bam*HI
Primer 4	*pyk*	CTCTCTGCAGATGCTCTGCTCAAGAAGTGC	*Pst*I
Primer 5	*pfk*	CTCTGGATCCATAAGATGGTCAGAGACAGT	*Bam*HI
Primer 6	*pfk*	CTCTGAGCTCAGTCAAGCCTAGGTCACAGT	*Sac*I
Primer 7	pCH	GGATCCGATATCCTGCAGGAG	
Primer 8	pCH	CTCGACCAACAGTTGCGCAGC	
Primer 9	*araR*	CTCTCTGCAGGCGCTCAATGCTTGACAGCG	*Pst*I
Primer 10	*araR*	CTCTAAGCTTCCGACGGCATCTACACCGAT	*Hin*dIII
Primer 11	*araR*	CTCTGAATTCTCGTGGAGGTTTCGCAGGAA	*Eco*RI
Primer 12	*araR*	CTCTCTGCAGGCTGCCAATGACCAGATGGC	*Pst*I

^a^ The restriction site overhangs used in the cloning procedure are underlined

### Sugar utilization by *C. glutamicum* strains

All wild-type and recombinant *C. glutamicum* strains were grown aerobically at 30 °C to late log phase in A medium, and then harvested by centrifugation (7000×*g*, 4 °C, 10 min). The cell pellets were subsequently washed with mineral salt BT medium. After the second wash, cells were inoculated into 100 mL of BT medium containing an appropriate concentration of sugars to an optical density at 600 nm (OD_600_) of 0.2. The resulting culture was incubated at 30 °C in a 500-mL baffled flask with constant agitation (170 rpm).

### Assay methods

Culture broth was centrifuged (15,000×*g*, 4 °C, 10 min), and the concentrations of d-glucose and l-arabinose in the supernatant were measured as follows. The concentration of l-arabinose and d-glucose was assayed enzymatically using an l-Arabinose/d-Galactose Assay kit (Megazyme, Wicklow, Ireland) and a Glucose CII test kit (Wako Pure Chemical Industries, Osaka, Japan), respectively, according to the manufacturer’s instructions. Cell mass was estimated by measuring the OD_600_ using a spectrophotometer (U-3010; Hitachi, Tokyo, Japan).

Major metabolites of the central metabolic pathways [e.g., glycolysis, the PPP, and tricarboxylic acid (TCA) cycle] were analyzed using an ion-pairing LC–MS/MS method [42]. Dried cell extracts were dissolved in 50 µL of MilliQ water for LC–MS/MS-based profiling and quantitation of 30 intracellular *C. glutamicum* metabolites. The following metabolites were analyzed: sugar phosphates [glucose-6-phosphate (G6P), fructose-6-phosphate (F6P), frucotose-1,6-bisphosphate (FBP), dihydroxyacetone phosphate (DHAP), glyceraldehyde-3-phosphate (GAP), 2- and 3-phosphoglycerate (2PG + 3PG), phosphoenolpyruvate (PEP) 6-phosphoglycerate (6PG), ribulose-5-phosphate (Rib5P), ribose-5-phosphate (R5P), X5P, erythrose-4-phosphate (E4P), and sedoheptulose-7-phosphate (S7P)]; organic acids [aconitate, citrate (Cit), fumarate (Fum), isocitrate (IsoCit), malate (Mal), oxaloacetate (OXA), 2-oxoglutarate (AKG), pyruvate, and succinate (Suc)]; nucleotides [adenosine diphosphate (ADP) and ATP]; and coenzymes [acetyl-CoA (AcCoA), oxidized and reduced nicotinamide adenine dinucleotide (NAD^+^ and NADH, respectively), and oxidized and reduced nicotinamide adenine dinucleotide phosphate (NADP^+^ and NADPH, respectively)]. Metabolites were quantified as described previously [43] using an Agilent 1200 series MS and Agilent 6460 with Jet Stream Technology LC–MS/MS system (Agilent Technologies, Waldbronn, Germany) equipped with a Maestro C18 column (2.1 × 150 mm, 3-µm particle size; Shimadzu, Kyoto, Japan).

### Extraction of metabolic intermediates

For quantitative metabolomics, *C. glutamicum* cells were subjected to cold methanol quenching [44] with a slight modification, as follows. A total of 15 mL of liquid culture at OD_600_ of 2.0 was withdrawn from medium and immediately sprayed into a 50-mL centrifugal tube (LMS Co., Ltd., Tokyo, Japan) containing 2.0 volumes of 40% (v/v) aqueous methanol at − 25 °C. After sampling, the content of each tube was immediately mixed by vortexing for 5 s to quench cellular metabolism and subsequently centrifuged (4000×*g*, 5 min, − 9 °C). After decanting of the supernatant, cell pellets were washed with 8 mL of 0.8% (w/v) NaCl at 4 °C and subsequently centrifuged again (4000×*g*, 5 min, − 9 °C). After decanting the supernatant again, the tubes containing the cell pellets were submerged directly into liquid nitrogen and subsequently stored at − 80 °C until metabolite extraction. For metabolite extraction, 3.0 mL of cold methanol (− 25 °C) containing (+)-camphor-10-sulfonic acid (18 μg/L) was added to each tube as an internal standard for quantitative LC–MC/MS analysis. The tubes were vortexed for 30 s, and the resulting cell suspensions were incubated at − 30 °C for 1 h, after which, 1.5 mL of the suspensions were transferred to 15-mL centrifuge tubes (LMS Co., Ltd.) containing 2.1 mL of chloroform and 1.5 mL of distilled water, which were then mixed by vortexing for 5 s. After centrifugation of the resulting suspensions at 15,000×*g* at 4 °C for 5 min, the upper phases were transferred to new tubes and then dried under vacuum. The dried samples were stored at − 80 °C until metabolite analysis.

### Statistics

Differences in sugar and metabolic intermediate concentrations and differences in cell density between the fermentation media were compared using the paired Student’s *t* test. A *p* value of < 0.05 was considered statistically significant.

## Results

### Simultaneous utilization of two sugars was suppressed in the presence of excess glucose

**Fig. 2:**
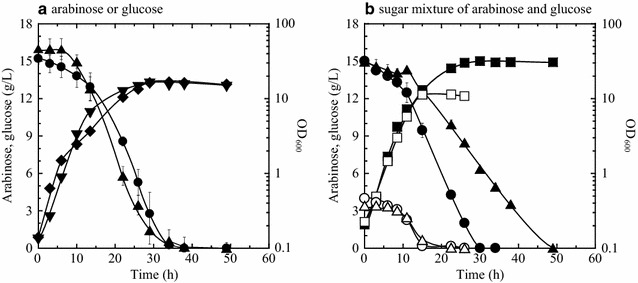
Consumption of l-arabinose and/or d-glucose by wild-type strain ATCC 31831 in mineral salt medium containing l-arabinose and d-glucose individually (**a**) or collectively (**b**). The wild-type strain was grown aerobically to late log phase in A medium (containing 20 g/L of l-arabinose and 20 g/L of d-glucose) and then inoculated to an initial OD_600_ of 0.2 into mineral salt BT medium. Using only a single carbon source (**a**), either l-arabinose or d-glucose was added to BT medium (15 g/L; solid symbols). Using two carbon sources (**b**), a mixture of l-arabinose and d-glucose was added to BT medium to final concentrations of either 3.6 g/L (open symbols) or 15 g/L (solid symbols). Cells were growth on either l-arabinose (reverse triangles) or d-glucose (diamonds) as single carbon sources, and also as a sugar mixture (squares). The concentrations of l-arabinose (empty triangles) and d-glucose (empty circles) are indicated. Data are plotted as the average ± standard deviation calculated from the results of triplicate individual experiments

Before mixed sugar utilization, sugar consumption and cell growth were investigated in mineral salt medium containing either d-glucose or l-arabinose as sole carbon source (15 g/L) in order to measure the rate of sugar consumption in the absence of a second carbon source. Wild-type strain ATCC 31831 was found to have an equivalent capacity for both l-arabinose metabolism and d-glucose metabolism in the absence of the second carbon source. l-Arabinose was consumed at a rate about equal to that of d-glucose (0.180 and 0.167 g/h/g dry cell weight (DCW), respectively), and the specific growth rate was higher on d-glucose than on l-arabinose [specific growth rate (*µ*) = 0.40 and 0.36 h^−1^, respectively] (Fig. 2a).

To examine the effect of concentration on the simultaneous utilization of two sugars, strain ATCC 31831 was aerobically grown in mineral salt medium containing l-arabinose and d-glucose as the carbon sources at concentrations of 3.6 or 15 g/L each. In the sugar mixture of l-arabinose and d-glucose at the low concentration (3.6 g/L each), both d-glucose and l-arabinose were consumed simultaneously at the same rate (Fig. 2b). In contrast, the high concentration sugar mixture (15 g/L each), elicited hindered l-arabinose metabolism due to excess d-glucose. After 30 h of cultivation, glucose was preferentially and completely consumed, while l-arabinose was consumed at a relatively lower rate with complete consumption at 49 h (Fig. 2b). The specific growth rate of the wild-type strain grown on the sugar mixture was significantly higher at the high concentration of 15 g/L each compared with the low concentration of 3.6 g/L each (*µ *= 0.35 and 0.31 h^−1^, respectively; *p* < 0.001).

Using only a single carbon source, the specific consumption rate of l-arabinose and d-glucose were comparable (Additional file 1: Table S1). In contrast, l-arabinose metabolism was apparently lower than d-glucose metabolism using the two carbon sources (Fig. 2b). At 23 h of cultivation, the specific consumption rates of l-arabinose and d-glucose using the two carbon sources were 0.043 and 0.070 g/h/g DCW, respectively (Fig. 2 and Additional file 1: Table S1). These results suggest that the limited carbon supply at low concentrations of d-glucose and l-arabinose (3.6 g/L each) restricts the growth of *C. glutamicum* and that excess glucose leads to preferential utilization of d-glucose over l-arabinose (15 g/L).

### Metabolome analysis of cells grown on glucose or arabinose

**Fig. 3:**
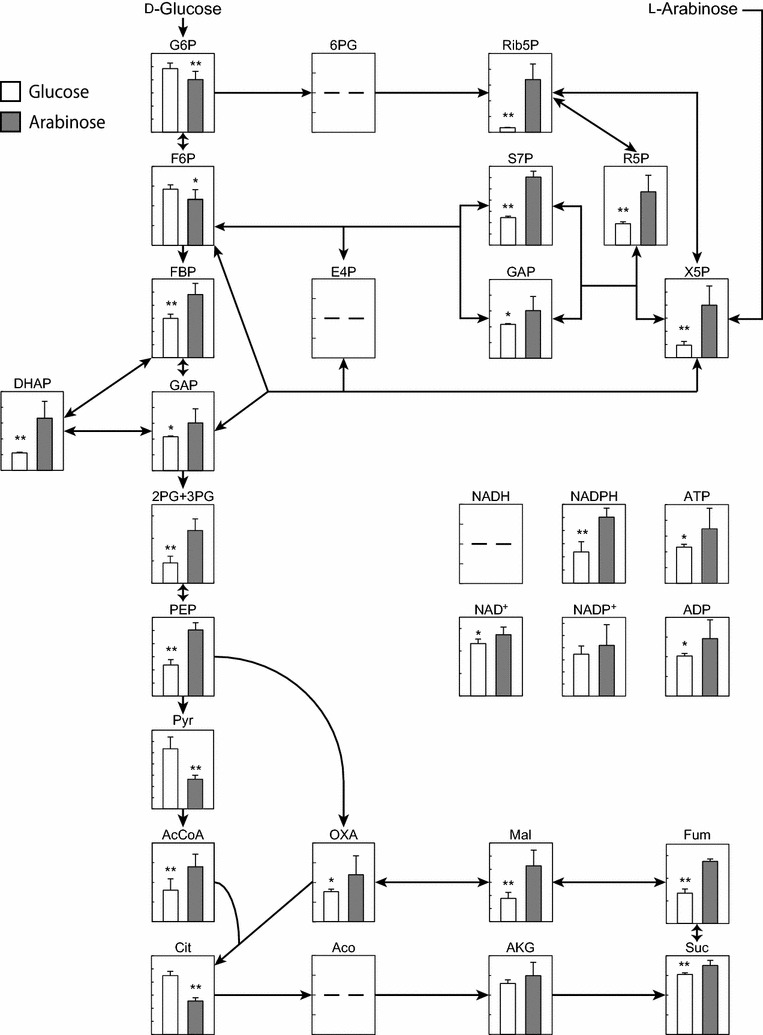
Metabolic profile of aerobically growing wild-type strain *C. glutamicum* ATCC 31831. Relative abundance of metabolic intermediates in *C. glutamicum* cells during aerobic growth in BT medium containing either d-glucose (open columns) or l-arabinose (gray columns) as the sole carbon source are shown. For the profiling experiment, the wild-type strain was grown aerobically to late log phase in A medium containing d-glucose (20 g/L) and l-arabinose (20 g/L), and the culture was then inoculated to an initial OD_600_ of 0.2 into BT medium containing either d-glucose (20 g/L) or l-arabinose (20 g/L) as the sole carbon source. The cells were harvested at mid log phase (OD_600_ = 1.5) and subjected to metabolic profiling. Data are presented as average ± standard deviation calculated from the results of triplicate individual experiments. Single and double asterisks indicate *p* values of < 0.05 and < 0.01, respectively

To identify the metabolic bottlenecks in the metabolism of l-arabinose, the metabolic profiles of wild-type *C. glutamicum* cells grown aerobically on either d-glucose or l-arabinose as the sole carbon and energy source were compared. Cells growing aerobically were harvested at exponential phase (OD_600_ = 1.5) and quenched immediately with liquid nitrogen for the extraction of metabolic intermediates. Consequently, the enzymatic activities of phosphofructokinase and pyruvate dehydrogenase, which convert F6P to FBP and pyruvate to AcCoA, respectively, were found to be major bottlenecks in d-glucose metabolism. As compared with cells grown on l-arabinose, the concentrations of G6P and F6P were significantly higher in cells grown on d-glucose, whereas the concentration of FBP was significantly lower (Fig. 3). In addition, the concentration of pyruvate was significantly higher in cells grown on d-glucose as compared with cells grown on l-arabinose, whereas the concentration of AcCoA was significantly lower. In contrast, the enzymatic activities of pyruvate kinase and citrate synthase, which convert PEP to pyruvate and AcCoA and OXA to Cit, respectively, were found to be major bottlenecks in l-arabinose metabolism. As compared with cells grown on d-glucose, the concentration of PEP was significantly higher in cells grown on l-arabinose, whereas the concentration of pyruvate was significantly lower. In addition, the concentrations of AcCoA and OXA were significantly higher and that of Cit significantly lower in cells grown on l-arabinose compared with cells grown on d-glucose. Furthermore, flux through the non-oxidative PPP was found to be higher during l-arabinose metabolism than during d-glucose metabolism. The concentrations of the PPP intermediates R5P, Rib5P, S7P, and X5P were significantly higher in cells grown on l-arabinose compared with cells grown on d-glucose.

### Metabolic engineering for simultaneous utilization of d-glucose and l-arabinose

To identify the critical bottlenecks in the simultaneous utilization of l-arabinose and d-glucose, three potential rate-limiting enzymes were selected for analysis based on metabolomic data (Fig. 3): citrate synthase, phosphofructokinase, and pyruvate kinase. Each of these enzymes was individually overexpressed in the wild-type strain. Pyruvate kinase or citrate synthase was overexpressed to eliminate major bottleneck in l-arabinose metabolism, whereas phosphofructokinase was overexpressed to eliminate the major bottleneck in d-glucose metabolism.

**Fig. 4:**
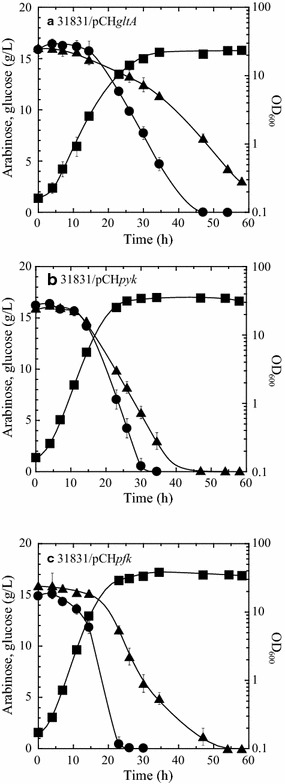
Utilization of a mixture of l-arabinose and d-glucose by aerobically growing cells of recombinant strains expressing citrate synthase (**a**), pyruvate kinase (**b**), or phosphofructokinase (**c**) genes. Recombinant strains were grown aerobically to late log phase in A medium (containing 20 g/L of l-arabinose and 20 g/L of d-glucose) and then inoculated to an initial OD_600_ of 0.2 into mineral salt BT medium containing l-arabinose and d-glucose (15 g/L each) as the carbon sources. Cell growth (squares) and the concentrations of l-arabinose (triangles) and d-glucose (circles) are indicated. Data are average ± standard deviation calculated from the results of triplicate individual experiments

Overexpression of citrate synthase reduced the consumption of both d-glucose and l-arabinose, and the specific growth rate of the recombinant strain declined by 40% compared with the wild-type strain (*µ* = 0.21 and 0.35 h^−1^, respectively) (Figs. 4a and 2b). Conversely, overexpression of pyruvate kinase (NCgl2008) reduced the time required for the complete consumption of l-arabinose without any effect on the time for d-glucose consumption, as compared to the wild-type strain (Figs. 4b and 2b). At 23 h of cultivation, the specific consumption rates of l-arabinose and d-glucose were 0.050 and 0.102 g/h/g DCW, respectively. The rate for l-arabinose consumption was 16% higher than that of the wild-type strain (Additional file 1: Table S1). In addition, the specific growth rate of the recombinant strain 31831/pCH*pyk* (*µ* = 0.28 h^−1^) was lower than that of the wild type strain. Overexpression of the endogenous pyruvate kinase isozyme (NCgl2809) did not effectively improve mixed sugar consumption (data not shown).

By contrast, overexpression of phosphofructokinase promoted preferential utilization of d-glucose over l-arabinose. As compared with the wild-type strain, strain 31831/pCH*pfk* increased d-glucose consumption but markedly reduced l-arabinose consumption, although the specific growth rate of the recombinant strain increase by 6% (*µ* = 0.33 h^−1^) (Figs. 4c and 2b). The recombinant strain 31831/pCH*pyk* exhibited 2.2-fold higher pyruvate kinase activity and a significantly decrease in the concentration gap between d-glucose and l-arabinose after 23 h of cultivation as compared with the wild-type strain (Table 3). Therefore, pyruvate kinase (NCgl2008) activity is likely a major bottleneck in the simultaneous utilization of l-arabinose and d-glucose.

**Table 3:** Utilization of d-glucose and l-arabinose and pyruvate kinase activity of aerobically grown wild-type strain, *araR*-deletion mutant, and recombinant strains expressing pyruvate kinase

Strain^a^	Sugar concentration (g/L)^b^		Δ Arabinose–glucose (g/L)^c^	Pyruvate kinase activity (µmol/min/mg protein)^d^
	d-Glucose	l-Arabinose		
Wild type	3.5 ± 1.5	9.2 ± 0.4	5.7 ± 1.2	0.54 ± 0.02
Δ*araR*	2.1 ± 1.5	8.4 ± 0.3	6.2 ± 1.2	0.45 ± 0.06
pCH*pyk*	7.8 ± 0.8	9.5 ± 0.8	1.7 ± 1.2	1.17 ± 0.04
Δ*araR*/pCH*pyk*	6.5 ± 1.3	7.1 ± 0.5	0.6 ± 0.8	1.61 ± 0.10

^a^ Recombinant strains were grown aerobically to late log phase in A medium containing 20 g/L of l-arabinose and 20 g/L of d-glucose and then inoculated to an initial OD_600_ of 0.2 into mineral salt BT medium containing l-arabinose and d-glucose (15 g/L each) as carbon sources

^b^ After 23 h of cultivation, the culture supernatant was collected by centrifugation and subjected to sugar analysis. Data shown are average ± standard deviation calculated from the results of triplicate individual experiments

^c^ Values were determined based on the concentration of d-glucose and l-arabinose after 23 h of cultivation. Data are average ± standard deviation calculated from the results of triplicate individual experiments

^d^ Pyruvate kinase activity was determined using crude cell extract prepared from cells grown for 23 h in mineral salt BT medium containing l-arabinose and d-glucose (15 g/L each) as carbon sources. Data are average ± standard deviation calculated from the results of triplicate analyses

### Effect of *araR* deletion in combination with increased pyruvate kinase

**Fig. 5:**
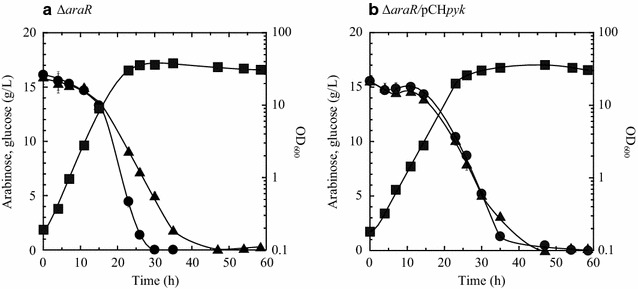
Utilization of a mixture of l-arabinose and d-glucose by the aerobically growing *araR*-deletion mutant (**a**) and its recombinant strain expressing pyruvate kinase (**b**). Both strains were grown aerobically to late log phase in A medium (containing 20 g/L of l-arabinose and 20 g/L of d-glucose) and then inoculated to an initial OD_600_ of 0.2 into mineral salt BT medium containing l-arabinose and d-glucose (15 g/L respectively) as the carbon sources. Cell growth (squares) and concentrations of l-arabinose (triangles) and d-glucose (circles) are indicated. Data are average ± standard deviation calculated from the results of triplicate individual experiments

Deletion of *araR* gene, which encodes a repressor of the l-arabinose-catabolizing genes *araA*, *araB*, and *araD* and the l-arabinose transporter gene *araE* (Fig. 1), leads to constitutive expression of these four genes [21]. To match the consumption rates of d-glucose and l-arabinose more closely, pyruvate kinase was overexpressed in the *araR*-deletion mutant (Δ*araR*). With overexpression of pyruvate kinase, the recombinant strain Δ*araR/*pCH*pyk* consumed l-arabinose and d-glucose at a comparable rate until 30 h of cultivation, whereas the Δ*araR* mutant utilized d-glucose preferentially after 17 h of cultivation (Fig. 5). Compared with the Δ*araR* mutant, strain Δ*araR/*pCH*pyk* exhibited significantly lower specific growth rates (μ = 0.29 and 0.21 h^−1^, respectively) and reduced d-glucose consumption rates. Compared with the Δ*araR* mutant at 23 h of cultivation, strain Δ*araR/*pCH*pyk* reduced the specific consumption rate of d-glucose (0.101 and 0.081 g/h/g DCW, respectively) and increased the specific consumption rate of l-arabinose (0.053 and 0.078 g/h/g DCW, respectively) (Additional file 1: Table S1). Meanwhile, total consumption rate of l-arabinose and d-glucose was comparable between both strains (approximately 0.16 g/h/g DCW). Consequently, the concentration gap between d-glucose and l-arabinose was reduced in culture with strain Δ*araR/*pCH*pyk*, which exhibited 3.6-fold higher pyruvate kinase activity as compared to strain Δ*araR* (Table 3). Thus, increased pyruvate kinase activity in combination with increased l-arabinose catabolism matched the consumption rates of l-arabinose and d-glucose in the high-concentration mixture of both carbon sources.

### Metabolic profiling of cells simultaneously utilizing d-glucose and l-arabinose

**Fig. 6:**
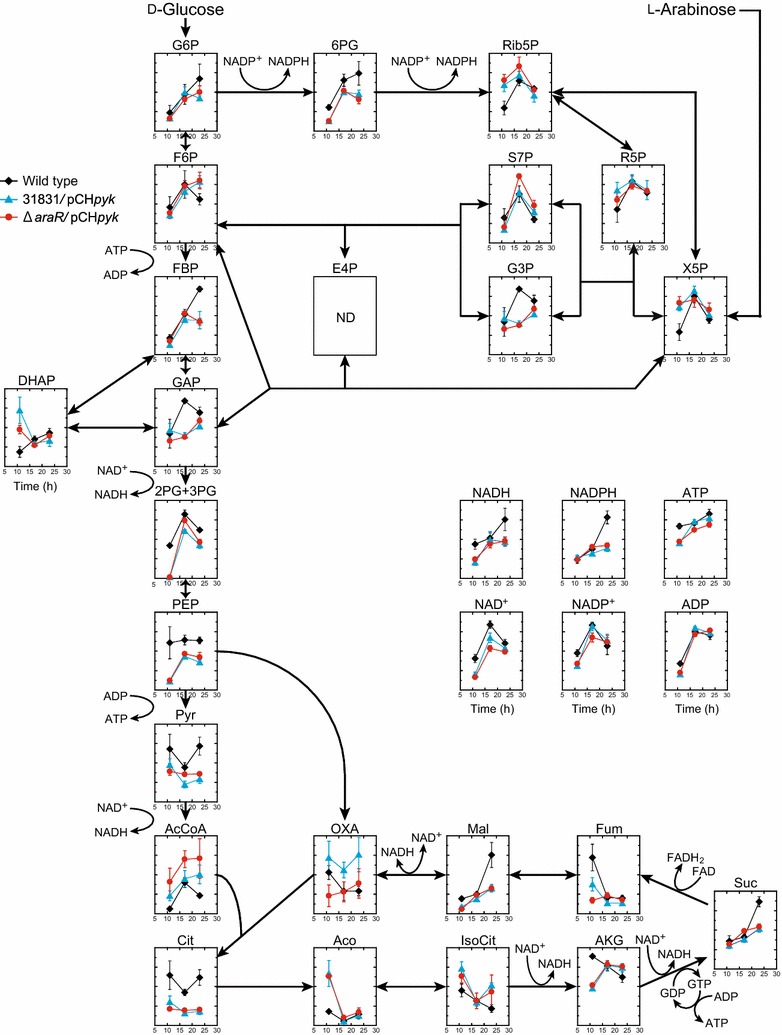
Time course metabolomics of aerobically growing wild-type *C. glutamicum* ATCC 31831 (black diamonds) and recombinant strains overexpressing pyruvate kinase in the absence or presence of *araR* deletion (31831/pCH*pyk*, blue triangles; Δ*araR*/pCH*pyk*, red circles). Relative abundances of metabolic intermediates in cells during aerobic growth in BT medium containing a mixture of d-glucose and l-arabinose are shown. The X-axis indicates cultivation time (h). For the profiling experiment, three strains were grown aerobically to late log phase in A medium containing 20 g/L of l-arabinose and 20 g/L of d-glucose, and the culture was then inoculated to an initial OD_600_ of 0.2 into BT medium containing a mixture of d-glucose and l-arabinose as the carbon sources (15 g/L each). The cells were harvested at 11, 17, and 23 h of cultivation and subsequently subjected to metabolome analysis. Data are presented as average ± standard deviation calculated from the results of triplicate individual experiments. The concentration of erythrose-4-phosphate was below the detection limit

To investigate differences in the metabolism of cells exhibiting preferential or simultaneous utilization of d-glucose in the presence of l-arabinose, transient states of metabolic profiles during consumption of mixtures of d-glucose and l-arabinose were probed by metabolome analysis. The wild-type strain was used as a control to demonstrate preferential utilization of d-glucose over l-arabinose, whereas the recombinant strain Δ*araR/*pCH*pyk* was used to examine simultaneous utilization. In addition, strain 31831/pCH*pyk* was used to investigate the effect of increased pyruvate kinase activity alone on the metabolic profile. Overall, increased pyruvate kinase activity had a marked impact on the metabolic profile, whereas synergetic effects involving *araR* deletion on the metabolic profile were relatively limited (Fig. 6).

In the oxidative PPP, levels of 6PG were significantly reduced by the overexpression of pyruvate kinase (Fig. 6). In the reductive PPP, by contrast, strain Δ*araR*/pCH*pyk* consuming d-glucose and l-arabinose simultaneously exhibited the highest levels of the l-arabinose catabolites Rib5P and X5P at 11 h of cultivation and retained high X5P levels throughout the observed period of 11–23 h, as compared with the wild-type strain. After 17 h of cultivation, S7P levels were significantly increased only in strain Δ*araR*/pCH*pyk*. No significant difference in expression of *tkt* (encoding transketolase) was observed between the wild-type and Δ*araR*/pCH*pyk* strains at 11 h of cultivation, whereas the expression of *tal* (encoding transaldolase) at 11 and 23 h was markedly higher in Δ*araR*/pCH*pyk* than in the wild-type strain (Additional file 1: Table S2).

l-Arabinose catabolites through the reductive PPP enter into glycolysis via F6P and GAP. No significant difference was observed in the level of the hexose phosphate F6P at 11 and 17 h of cultivation, while the level of the triose phosphate GAP was reduced by the overexpression of pyruvate kinase at 17 and 23 h of cultivation (Fig. 6). Levels of the glycolytic intermediates PEP and pyruvate were markedly affected by pyruvate kinase activity. Throughout the observed period of 11–23 h, overexpression of pyruvate kinase reduced PEP and pyruvate levels significantly, as compared with the wild-type strain. Conversely, AcCoA levels increased following overexpression of pyruvate kinase and increased further in combination with increased l-arabinose catabolism (Δ*araR*/pCH*pyk*). No significant differences were observed in the expression levels of genes encoding key enzymes in d-glucose uptake and glycolysis, except for reduced levels of *ptsI* in strain Δ*araR*/pCH*pyk* at 11 h of cultivation (Additional file 1: Table S2). Thus, strain Δ*araR*/pCH*pyk* appeared to enhance the metabolic flux of triose phosphates, leading to elevated AcCoA levels during simultaneous sugar utilization.

Overexpression of pyruvate kinase also affected the profiles of the TCA cycle intermediates. Levels of Cit were significantly reduced by the overexpression of pyruvate kinase. Conversely, levels of the Cit precursors AcCoA and OXA varied depending on strain (Fig. 6), although the concentrations of OXA were relatively low (Additional file 1: Figs S1 and S2). Simultaneous utilization of l-arabinose and d-glucose appeared to attenuate reductive reactions dependent on NAD^+^ and FAD as cofactors. At 11 h of cultivation, the two recombinant strains showed significantly increased aconitate levels and significantly reduced AKG levels, as compared with the wild-type strain. In addition, both recombinant strains showed significantly lower fumarate levels but comparable succinate levels, as compared with the wild-type strain.

Simultaneous utilization of l-arabinose and d-glucose affected energy state and redox balance in the cell. Strain Δ*araR*/pCH*pyk* has lower-energy and more-oxidative (low ATP/ADP and NADPH/NADP^+^ ratios) states during simultaneous utilization of d-glucose and l-arabinose (23 h), compared with the wild-type strain preferentially utilizing d-glucose. At 23 h of cultivation, the ATP/ADP, NADH/NAD^+^, and NADPH/NADP^+^ ratios in the wild-type strain were 0.757, 0.0016, and 1.287, respectively, whereas those in the strain Δ*araR*/pCH*pyk* were 0.579, 0.0013, and 0.633, respectively. Throughout the observed period of 17–23 h of simultaneous sugar utilization, ATP levels were lower in strain Δ*araR*/pCH*pyk* relative to strain 31831/pCH*pyk*.

## Discussion

Metabolome analysis has become a powerful tool for comprehensively investigating metabolic profiles [45]. In this study, metabolome analysis was used to identify potential bottlenecks in *C. glutamicum* metabolism of d-glucose and l-arabinose (Fig. 3) via overexpression of predicted rate-limiting enzymes (Fig. 4). Combining metabolome analysis and metabolic engineering enabled the development of an engineered metabolic pathway in which d-glucose and l-arabinose can be utilized simultaneously in the presence of excess d-glucose (Fig. 5). In a previous study, stepwise overexpression of four genes encoding glycolytic enzymes improved glucose metabolism [46]. In the present study, the results of metabolome analysis revealed that manipulation of only two genes is necessary for simultaneous utilization of d-glucose and l-arabinose at the same rate. Several laboratories have investigated the metabolic status of *C. glutamicum* with the ultimate goal of improving d-glucose consumption and fermentation [35, 47, 48] however optimization of l-arabinose consumption has not yet been reported. The present study of *C. glutamicum* is the first to demonstrate that metabolome analysis can provide insight into bottlenecks that can be overcome by the rational design metabolic pathways, leading to optimization of simultaneous consumption rates of d-glucose and l-arabinose.

Previous strategies to avoid CCR have depended on inactivation of the PTS, resulting in markedly reduced rates of d-glucose utilization in both *E. coli* and *C. glutamicum* [49, 50]. In contrast to the previous approach, the present strategy preserved the indigenous PTS with a high efficiency for d-glucose uptake, and the resulting metabolically engineered strains utilized d-glucose without any decline in consumption rate (Fig. 5). The engineered strain Δ*araR*/pCH*pyk* utilized d-glucose and l-arabinose simultaneously at exact the same rate (Fig. 5). Previous PTS^‒^ mutants of both *E. coli* and *C. glutamicum* demonstrated reduced or incomplete sugar utilization in the presence of multiple sugars [13, 50]. Therefore, the present PTS-conserved strategy is an novel metabolic engineering approach that avoids CCR without any decline in sugar utilization rate and does not require adaptive laboratory evolution selection that could result in unexpected mutations in the genome and hinder further metabolic engineering to develop a microbial platform [51].

Pyruvate kinase activity was a major bottleneck in l-arabinose metabolism, and overexpression of the enzyme was found to be a prerequisite for simultaneous utilization of d-glucose and l-arabinose (Fig. 5 and Additional file 1: Table S1). The increased pyruvate kinase activity increased the specific consumption rate of l-arabinose yet only moderately reduced the specific consumption rate of d-glucose, particularly in combination with *araR* deletion (Figs. 4 and 5 and Additional file 1: Table S1). PEP, the substrate of pyruvate kinase, represents the switch point in the carbon flux distribution in bacteria [52]. For pentose metabolism, the flux through PEP to pyruvate in glycolysis depends completely on pyruvate kinase activity [53]. For d-glucose metabolism, by contrast, pyruvate kinase serves as a bypass in the flux, as 50% of the PEP in the glycolysis is converted into pyruvate by the PTS [14]. Thus, increased pyruvate kinase activity has two physiologic implications for multiple sugar consumption: promotion of pentose flux into glycolysis and slight depression of d-glucose uptake mediated by PTS owing to reduce available PEP for the PTS (Fig. 1).

As the specific consumption rate of l-arabinose increased, both specific growth rates and intracellular ATP levels were reduced (Fig. 6 and Additional file 1: Table S1). Pyruvate kinase-dependent pentose metabolism consumes more ATP than d-glucose metabolism mediated by PTS [54]. Thus, the more l-arabinose consumption leads to the less ATP generation. In the present study, ATP levels were lower in strain Δ*araR/*pCH*pyk* than the wild-type strain (Fig. 6). Consequently, reduced ATP levels led to reduced specific growth rate of recombinant strains overexpressing pyruvate kinase, particular in strain Δ*araR/*pCH*pyk* (Additional file 1: Table S1). Total sugar consumption rate of the recombinant strain Δ*araR/*pCH*pyk* was not over that of the wild-type strain using only a single carbon source of either d-glucose or l-arabinose, despite the specific consumption rate of l-arabinose was 1.8-fold higher in strain Δ*araR/*pCH*pyk* than the wild-type strain (Additional file 1: Table S1). Expression levels of key genes for l-arabinose and d-glucose were comparable or higher in strain Δ*araR/*pCH*pyk* than the wild-type strain, except of *ptsI* encoding enzyme I for PTS (Additional file 1: Table S2 and Fig. 1). These result support the hypothesis that the consumption of hexose and pentose mixtures is regulated metabolically rather than transcriptionally by intracellular conditions, such as energy state, redox balance, concentrations of metabolic intermediates or the capacity of glycolysis, as reported previously [47, 55–59].

Deletion of *araR* in combination with overexpression of pyruvate kinase was crucial to continue simultaneous utilization of d-glucose and l-arabinose after 17 h of cultivation (Fig. 5). Furthermore, the metabolome analysis revealed synergetic effects of the combined approach on metabolic profiles with elevated levels of l-arabinose catabolites (X5P, Rib5P, and S7P) and AcCoA in strain Δ*araR/*pCH*pyk* (Fig. 6). The *araR*-deletion mutant expressed l-arabinose–catabolizing genes constitutively [21], and increased expression of these genes (*araA*, *araB*, and *araE*) was observed in strain Δ*araR* when utilizing d-glucose and l-arabinose (Additional file 1: Table S2). The transporter AraE also uptakes d-xylose with a high efficiency [27]. Thus, the engineered pathway of strain Δ*araR/*pCH*pyk* can be also applied to co-utilization of d-glucose and d-xylose, which comprise a major fraction of lignocellulosic feedstocks [2]. In the PPP, expression levels of *tal* after 11 and 23 h were markedly higher in strain Δ*araR*/pCH*pyk* than the wild-type strain (Additional file 1: Table S2). Increased *tal* expression was also observed during d-xylose metabolism in *E. coli* [53]. It is therefore hypothesized that increased entry of l-arabinose catabolites enhances flux through the non-oxidative PPP in strain Δ*araR/*pCH*pyk*, resulting in matching of the rates of simultaneous utilization of d-glucose and l-arabinose.

With overexpression of pyruvate kinase, intracellular AcCoA levels were significantly elevated, while Cit levels were significantly reduced (Fig. 6). The increment of AcCoA in the strain overexpressing pyruvate kinase was markedly higher in combination with *araR* disruption, indicating that l-arabinose catabolism and subsequent metabolism of triosephosphates was enhanced in strain Δ*araR*/pCH*pyk*. Citrate synthase activity controls carbon allocation between anabolism and catabolism in *C. glutamicum* for l-lysine production [60] and also limits growth of *E. coli* on another pentose, d-xylose [61]. In addition, the present metabolome analysis revealed that citrate synthase activity is a major bottleneck in l-arabinose metabolism (Fig. 3). Our results indicate that citrate synthase activity becomes the new bottleneck in the engineered pathway for the simultaneous utilization of d-glucose and l-arabinose.

## Conclusions

Based on the results of metabolome analysis, a metabolic pathway was designed and engineered to enable matching of the rates of simultaneous utilization of d-glucose and l-arabinose by *C. glutamicum*. The metabolome analysis revealed potential bottlenecks to be eliminated for co-metabolism of d-glucose and l-arabinose and provided the basis for the rational design of an engineered metabolic pathway. The manipulation of only two genes in combination with overexpression of pyruvate kinase and the deletion of *araR* enabled simultaneous and complete utilization of d-glucose and l-arabinose. The designed pathway for robust simultaneous sugar utilization is quite simple and unique, as inactivation of the PTS to avoid CCR and subsequent adaptive mutation to recover the d-glucose utilization rate are unnecessary. Such a simple strategy has not been reported, even in *E. coli*. Thus, the rational design strategy developed in the present study can be applied to develop a microbial platform for microbial production, as adaptive mutation that could produce unexpected DNA damage in the host genome is no longer necessary.

## Additional file

**Additional file 1: Table S1.** Specific growth rates and specific sugar consumption rates by recombinant strains and the wild-type strain of *C. glutamicum* ATCC 31831. **Table S2.** Relative expression levels of genes in the *ara* cluster and central metabolic pathway in aerobically grown wild-type and *araR*-deletion mutants with/without *pyk* overexpression (Δ*araR* and Δ*araR*/pCH*pyk*). **Fig. S1.** Retention time comparison of liquid chromatography–mass spectrometry/mass spectrometry total ion current chromatograms of oxaloacetate (OXA) in *C. glutamicum* cells grown aerobically in BT medium containing sugar mixture of d-glucose and l-arabinose (15 g/L each). The authentic standards of OXA at the concentration of 2, 10, and 20 μM (a, b, c) as well as their identified counterparts in *C. glutamicum* cells of the wild-type (d), and recombinant strains of 31831/pCH*pyk* (e) and Δ*araR*/pCH*pyk* (f). OXA was identified based on retention time in chromatography and its mass spectrum. Concentration of OXA was determined with peak area of its multiple reaction monitoring (MRM) transition (131.0>87.1) in LC-MS/MS. **Fig. S2.** Mass spectra of identified oxaloacetate (OXA) in *C. glutamicum* cells grown aerobically in BT medium containing sugar mixture of d-glucose and l-arabinose (15 g/L each). The authentic standards of OXA at the concentration of 2, 10, and 20 μM (A) as well as their identified counterparts in *C. glutamicum* cells of the wild-type (d), and recombinant strains of 31831/pCH*pyk* (e) and Δ*araR*/pCH*pyk* (f). OXA was identified based on retention time in chromatography and its mass spectrum acquired by the targeted multiple reaction monitoring (MRM) transition (131.0>87.1). **Table S3.** Oligonucleotides used for qPCR in this study.

## Abbreviations

AcCoA: acetyl CoA
ADP: adenosine 5′-diphosphate
AKG: 2-oxoglutarate
ATP: adenosine 5′-triphosphate
CCR: carbon catabolite repression
Cit: citrate
E4P: erythrose-4-phosphate
FBP: fructose-1,6-bisphosphate
F6P: fructose-6-phosphate
GAP: glycelaldehyde-3-phosphate
G6P: glucose-6-phosphate
IsoCit: isocitrate
NAD^+^: nicotinamide adenine dinucleotide oxidized
NADH: nicotinamide adenine dinucleotide reduced
NADP^+^: nicotinamide adenine dinucleotide phosphate oxidized
NADPH: nicotinamide-adenine dinucleotide phosphate reduced
OXA: oxaloacetate
PEP: phosphoenolpyruvate
6PG: 6-phosphogluconate
PPP: pentose phosphate pathway
PTS: phosphotransferase system
Rib5P: ribulose-5-phosphate
R5P: ribose-5-phosphate
S7P: sedoheptulose-7-phosphate
TCA: tricarboxylic acid
X5P: xylulose-5-phosphate
