# Supplementary material for: Metabolome analysis-based design and engineering of a metabolic pathway in Corynebacterium glutamicum to match rates of simultaneous utilization of d-glucose and l-arabinose
Source: Microb Cell Fact. 2018 May 17;17:76. doi: 10.1186/s12934-018-0927-6 (PMC5956887; doi:10.1186/s12934-018-0927-6)
Supplement: Supplementary file 1 — Additional file 1: Table S1. Specific growth rates and specific sugar consumption rates by recombinant strains and the wild-type strain of C. glutamicum ATCC 31831. Table S2. Relative expression levels of genes in the ara cluster and central metabolic pathway in aerobically grown wild-type and araR-deletion mutants with/without pyk overexpression (ΔaraR and ΔaraR/pCHpyk). Fig. S1. Retention time comparison of liquid chromatography–mass spectrometry/mass spectrometry total ion current chromatograms of oxaloacetate (OXA) in C. glutamicum cells grown aerobically in BT medium containing sugar mixture of d-glucose and l-arabinose (15 g/L each). The authentic standards of OXA at the concentration of 2, 10, and 20 μM (a, b, c) as well as their identified counterparts in C. glutamicum cells of the wild-type (d), and recombinant strains of 31831/pCHpyk (e) and ΔaraR/pCHpyk (f). OXA was identified based on retention time in chromatography and its mass spectrum. Concentration of OXA was determined with peak area of its multiple reaction monitoring (MRM) transition (131.0>87.1) in LC-MS/MS. Fig. S2. Mass spectra of identified oxaloacetate (OXA) in C. glutamicum cells grown aerobically in BT medium containing sugar mixture of d-glucose and l-arabinose (15 g/L each). The authentic standards of OXA at the concentration of 2, 10, and 20 μM (A) as well as their identified counterparts in C. glutamicum cells of the wild-type (d), and recombinant strains of 31831/pCHpyk (e) and ΔaraR/pCHpyk (f). OXA was identified based on retention time in chromatography and its mass spectrum acquired by the targeted multiple reaction monitoring (MRM) transition (131.0>87.1). Table S3. Oligonucleotides used for qPCR in this study. [file 12934_2018_927_MOESM1_ESM.pdf]

Metabolome analysis–based design and engineering of a metabolic pathway in *Corynebacterium glutamicum* to match rates of simultaneous utilization of D-glucose and L-arabinose

Hideo Kawaguchi[a], Kumiko Yoshihara[a], Kiyotaka Y. Hara[a,b], Tomohisa Hasunuma[a], Chiaki Ogino[c], Akihiko Kondo[a,c,d,]*

[a] Graduate School of Science, Technology and Innovation, Kobe University, 1-1 Rokkodai, Nada, Kobe 657-8501, Japan

[b] Department of Environmental and Life Sciences, School of Food and Nutritional Sciences, University of Shizuoka, 52-1 Yada, Suruga, Shizuoka 422-8526, Japan

[c] Department of Chemical Science and Engineering, Graduate School of Engineering, Kobe University, 1-1 Rokkodai, Nada, Kobe 657-8501, Japan

[d] Biomass Engineering Research Division, RIKEN, 1-7-22 Suehiro, Turumi, Yokohama, Kanagawa 230-0045, Japan

***Correspondence:** Akihiko Kondo

Graduate School of Science, Technology and Innovation, Kobe University

1-1 Rokkodai, Nada, Kobe 657-8501, Japan

E-mail: akondo@kobe-u.ac.jp

Tel: +81-78-803-6192

Fax: +81-78-803-6192

Table S1 Specific growth rates and specific sugar consumption rates by recombinant strains and the wild-type strain of *C. glutamicum* ATCC 31831

Strain	Carbon source[a]	Specific growth rate (h^{-1})	Specific consumption rate (g/h/g dry cell weight)[e]		Total amounts of sugar consumed (g/L)[e]
			Glucose	Arabinose	
Wild-type	Arabinose	0.36[b]	—	0.180[b]	9.2[b]
Wild-type	Glucose	0.40[b]	0.167[b]	—	6.4[b]
Wild-type	Mixture of arabinose and glucose	0.35[b]	0. 070[b]	0.043[b]	17.3[b]
31831/pCH*pyk*	Mixture of arabinose and glucose	0.28[c]	0.102[c]	0.050[c]	12.7[c]
Δ*araR*	Mixture of arabinose and glucose	0.29[d]	0.101[d]	0.053[d]	19.5[d]
Δ*araR*/pCH*pyk*	Mixture of arabinose and glucose	0.21[d]	0.081[d]	0.078[d]	16.4[d]

[a]BT medium containing both D-glucose and/or L-arabinose (15 g/L each) was used for aerobic cell growth.

[b]Values were determined based on data shown in Fig. 2.

[c]Values were determined based on data shown in Fig. 4.

[d]Values were determined based on data shown in Fig. 5.

[d]Values were determined from data at 23 h of cultivation.

Table S2 Relative expression levels of genes in the *ara* cluster and central metabolic pathway in aerobically grown wild-type and *araR*-deletion mutants with/without *pyk* overexpression (Δ*araR* and Δ*araR*/pCH*pyk*)

		Relative abundance[b, b]					
	Strain[a]	Wild type		Δ*araR*		Δ*araR*/pCH*pyk*	
Target gene[d]	Time (h)	11	23	11	23	11	23
araA		1.0 ± 0.26	0.84 ± 0.43<br>(0.590)	1.76 ± 0.12<br>(0.012)	2.90 ± 0.40<br>(0.007)	1.71 ± 0.17<br>(0.019)	3.52 ± 0.16<br>(0.001)
araB		1.0 ± 0.14	0.99 ± 0.48<br>(0.969)	1.58 ± 0.11<br>(0.014)	1.60 ± 0.46<br>(0.221)	1.55 ± 0.34<br>(0.133)	4.35 ± 2.04<br>(0.147)
araE		1.0 ± 0.25	0.65 ± 0.31<br>(0.178)	1.35 ± 0.28<br>(0.196)	1.25 ± 0.47<br>(0.498)	1.38 ± 0.23<br>(0.143)	1.63 ± 0.95<br>(0.341)
ptsG		1.0 ± 0.31	0.45 ± 0.10<br>(0.092)	0.84 ± 0.10<br>(0.373)	0.54 ± 0.17<br>(0.072)	1.03 ± 0.41<br>(0.995)	0.53 ± 0.13<br>(0.130)
ptsI		1.0 ± 0.06	1.28 ± 0.56<br>(0.435)	1.03 ± 0.19<br>(0.844)	1.63 ± 0.57<br>(0.132)	0.69 ± 0.03<br>(0.001)	1.99 ± 1.21<br>(0.230)
pfk		1.0 ± 0.02	0.67 ± 0.23<br>(0.078)	0.99± 0.23<br>(0.961)	1.60 ± 0.39<br>(0.058)	1.24 ± 0.15<br>(0.051)	0.86 ± 0.11<br>(0.098)
gapA		1.0 ± 0.13	0.46 ± 0.37<br>(0.157)	1.02 ± 0.17<br>(0.898)	1.28 ± 0.58<br>(0.580)	1.23 ± 0.13<br>(0.151)	0.80 ± 0.01<br>(0.158)
pyk		1.0 ± 0.14	1.03 ± 0.43<br>(0.925)	1.25 ± 0.08<br>(0.055)	4.13 ± 1.60<br>(0.028)	8.16 ± 2.17<br>(0.003)	3.64 ± 3.16<br>(0.214)
tal		1.0 ± 0.10	0.73 ± 0.26<br>(0.171)	1.45 ± 0.28<br>(0.060)	2.09 ± 0.52<br>(0.025)	1.61 ± 0.42<br>(0.059)	2.22 ± 0.35<br>(0.009)
tkt		1.0 ± 0.28	0.27 ± 0.11<br>(0.013)	0.79 ± 0.21<br>(0.304)	0.90 ± 0.01<br>(0.572)	1.38 ± 0.59<br>(0.406)	0.64 ± 0.29<br>(0.236)

[a]Cells were grown aerobically to late log phase in A medium containing D-glucose and L-arabinose (20 g/L each) and subsequently inoculated to an initial OD_{600} of 0.2 into BT medium containing both D-glucose and L-arabinose (15 g/L each).

[b]RNA was isolated from aerobically grown cells of the wild-type strain and *araR* deletion mutants with or without *pyk* overexpression harvested after 11 and 23 h of cultivation. The amount of each transcript was subsequently determined using quantitative RT-PCR and expressed relative to the amount obtained for the wild-type strain harvested after 11 h of cultivation. Data are average ± standard deviation calculated from the results of triplicate measurements.

[c]Values in parentheses indicate *p* values for comparison with the wild-type strain harvested after 11 h of cultivation.

[d]Abbreviations: *araA*, L-arabinose isomerase; *araB*, L-ribulokinase; *araE*, L-arabinose transporter; *ptsI*, enzyme I of PTS; *gapA*, glycelaldehyde-3-phosphate dehydrogenase; *pfk*, phosphosfructokinase; *ptsG*, D-glucose PTS transporter; *pyk*, pyruvate kinase (NCgl2008); *tal*, transalderase; *tkt*, transketolase.

Figure captions

Fig. S1 Retention time comparison of liquid chromatography–mass spectrometry/mass spectrometry total ion current chromatograms of oxaloacetate (OXA) in *C. glutamicum* cells grown aerobically in BT medium containing sugar mixture of D-glucose and L-arabinose (15 g/L each). The authentic standards of OXA at the concentration of 2, 10, and 20 μM (a, b, c) as well as their identified counterparts in *C. glutamicum* cells of the wild-type (d), and recombinant strains of 31831/pCH*pyk* (e) and Δ*araR*/pCH*pyk* (f). OXA was identified based on retention time in chromatography and its mass spectrum. Concentration of OXA was determined with peak area of its multiple reaction monitoring (MRM) transition (131.0>87.1) in LC-MS/MS.

Fig. S2 Mass spectra of identified oxaloacetate (OXA) in *C. glutamicum* cells grown aerobically in BT medium containing sugar mixture of D-glucose and L-arabinose (15 g/L each). The authentic standards of OXA at the concentration of 2, 10, and 20 μM (A) as well as their identified counterparts in *C. glutamicum* cells of the wild-type (d), and recombinant strains of 31831/pCH*pyk* (e) and Δ*araR*/pCH*pyk* (f). OXA was identified based on retention time in chromatography and its mass spectrum acquired by the targeted multiple reaction monitoring (MRM) transition (131.0>87.1).

Fig. S1

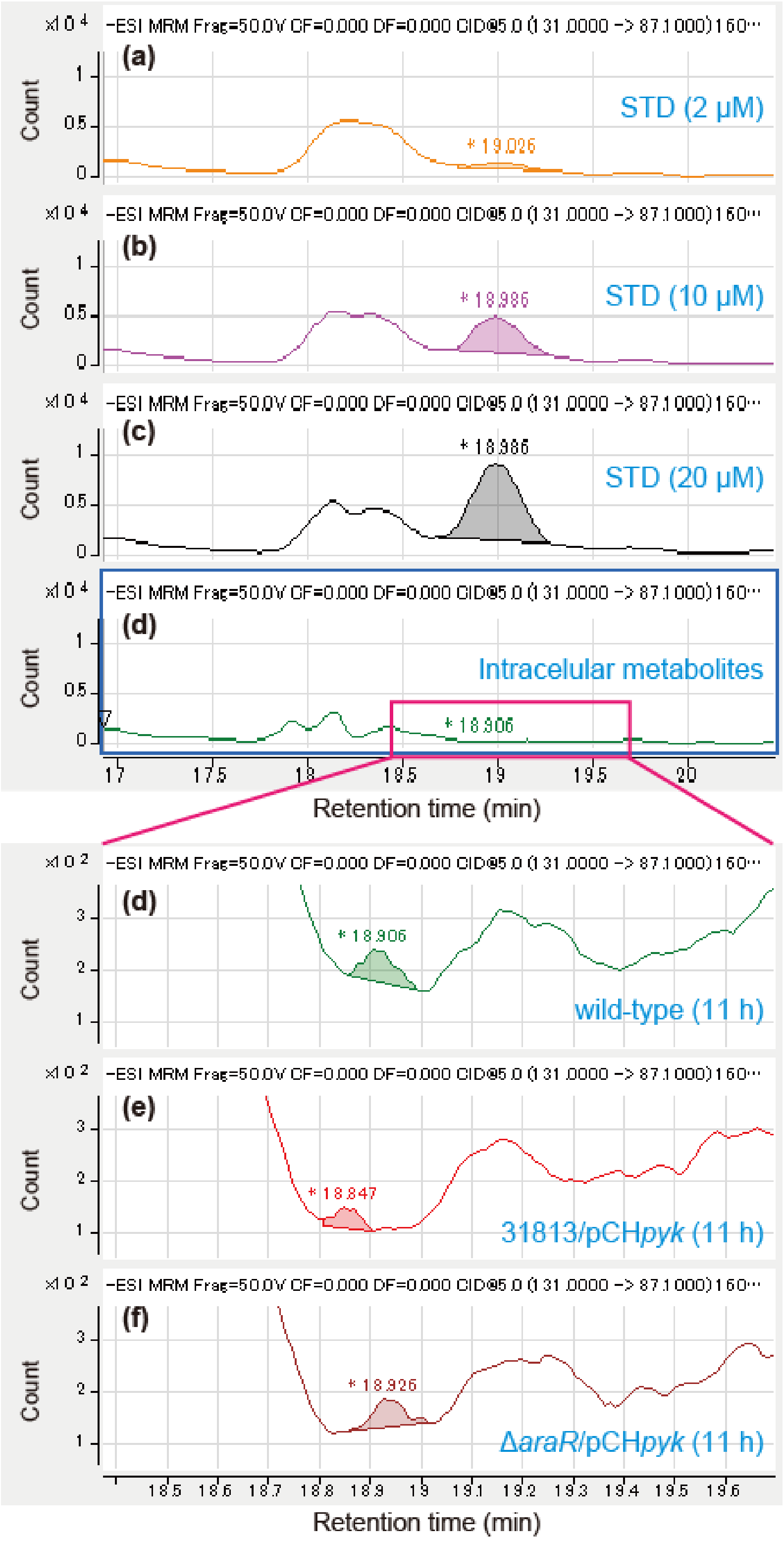
x10 4
-ESI MRM Frag=50.0V CF=0.000 DF=0.000 CID@5.0 (131.0000 -> 87.1000)160...
(a)
STD (2 μM)
* 19.026
(b)
STD (10 μM)
* 18.986
(c)
STD (20 μM)
* 18.986
(d)
Intracelular metabolites
* 18.906
Count
Retention time (min)
x10 2
(d)
* 18.906
wild-type (11 h)
(e)
* 18.847
31813/pCHpyk (11 h)
(f)
* 18.926
ΔaraR/pCHpyk (11 h)
Count
Retention time (min)

Fig. S2

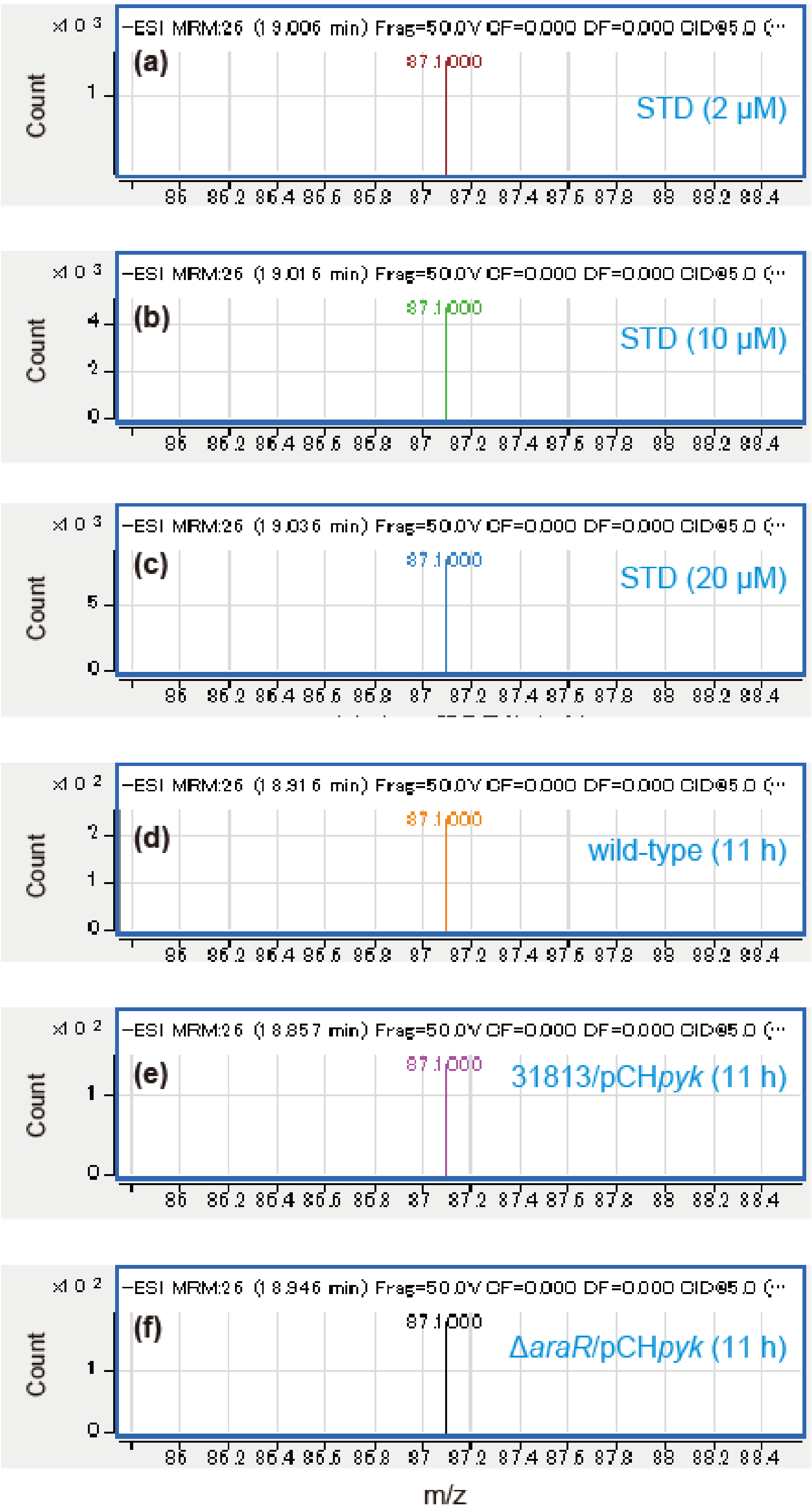
(a)
STD (2 μM)
(b)
STD (10 μM)
(c)
STD (20 μM)
(d)
wild-type (11 h)
(e)
31813/pCH*pyk* (11 h)
(f)
Δ*araR*/pCH*pyk* (11 h)
87.1000
Count
m/z

Materials and Methods

Quantitative RT-PCR analysis

Wild-type and recombinant (Δ*araR* and Δ*araR*/pCH*pyk*) strains of *C. glutamicum* were grown aerobically in BT medium containing D-glucose and L-arabinose (15 g/L each). Total RNA was extracted from growing cells using an RNeasy Mini kit (Qiagen, Hilden, Germany) as previous described (Kawaguchi et al., 2008). Total RNA isolated from the cells was used for quantitative reverse transcriptase PCR (qPCR) analysis to determine mRNA levels in wild-type and recombinant cells after 11 and 23 h of cultivation compared with levels in wild-type cells after 11 h of cultivation. qPCR was performed using QuantStudio 3 (Thermo Fisher Scientific, Waltham, MA) and PowerUp SYBR Green Master Mix (Thermo Fisher Scientific) according to the manufacturer's instructions. Oligonucleotides used for qPCR analysis are listed in Table S3 (primers 13 through 36). The comparative cycle threshold method was used to quantify relative expression. Cycle threshold values were computed by determining the average of triplicate values.

Table S3 Oligonucleotides used for qPCR in this study

Name	Target gene	Sequence (5'-3')
primer 13	*araA*	GCGCCAATGACAACGTCAT
primer 14	*araA*	TCATTAGCCTGGGTGTGAAGGT
primer 15	*araB*	TCGAACTGGATCGAGCTCTT
primer 16	*araB*	GTAGACCTCGTCGATACTGTTG
primer 17	*araD*	GATCATCGGTGACGACTCT
primer 18	*araD*	GTGGTTCTTCATCAGCACA
primer 19	*araE*	TAGCGTCCGCTGAATAGGGT
primer 20	*araE*	GCCATCGATTCCGAGCTCAA
Prime r21	*ptsG*	TATCCCATTGCTCTACCCA
primer 22	*ptsG*	CCCTGAATGAAGTCGTAACC
primer 23	*ptsI*	CGAACTGTGCTTCCTTTCC
primer 24	*ptsI*	ACAACGACCTTGGACTCT
primer 25	*pfk*	CACCGTTATTCCAGAAGTACC
primer 26	*pfk*	GACGATAATGCCGTACTTCTC
primer 27	*gapA*	GCAGTCAACATCGTTCCT
primer 28	*gapA*	TAACTGGAACGCGAAGTG
primer 29	*pyk*	GGACTTTATTGCACTGTCCTTC
primer 30	*pyk*	CTTGGCGATCACAGGAACA
primer 31	*tal*	TCCAAGATCCACTCTGTGGCTT
primer 32	*tal*	AAAGCCTCATCGGATCCGATTG
primer 33	*tkt*	CATCCTCAACGGCATTTCCCT
primer 34	*tlt*	CCATGAGAGCTGCAAGACGAA
primer 35	16S rRNA	CAGGTCTCTGGGCAGTAACTGA
primer 36	16S rRNA	CGTTTACGGCATGGACTACCA

Reference

Kawaguchi H, Sasaki M, Vertès AA, Inui M, Yukawa H. Engineering of an L-arabinose metabolic pathway in *Corynebacterium glutamicum*. Appl Microbiol Biotechnol. 2008;77:1053–62. doi:10.1007/s00253-007-1244-x.
